# Incidence and risk factors for postplacental intrauterine device expulsion in a Brazilian hospital

**DOI:** 10.1002/ijgo.70644

**Published:** 2025-11-04

**Authors:** Silvana A. Giovanelli, Maria Regina Torloni, Cristina A. F. Guazzelli

**Affiliations:** ^1^ Obstetrics Department Universidade Federal de São Paulo (UNIFESP) Escola Paulista de Medicina São Paulo SP Brazil

**Keywords:** copper, expulsion, intrauterine devices, postpartum period, risk factors

## Abstract

**Objective:**

This study assessed the incidence of and risk factors associated with expulsion of copper intrauterine devices inserted in the immediate postpartum period at a public hospital in Brazil.

**Methods:**

This retrospective cohort study included women who had a copper intrauterine device (IUD) inserted immediately after delivery at a teaching hospital and returned for follow‐up within 12 months. The primary outcome was IUD expulsion (partial or complete) overall and by mode of delivery. Univariate and multivariate logistic regression analyses were conducted to identify independent risk factors for expulsion. Results are presented as adjusted odds ratios (aOR) with 95% confidence intervals (CI).

**Results:**

Among the 543 participants, the overall expulsion rate was 14.7%, with significantly higher rates for placement after vaginal delivery (22.8%) compared to cesarean section (5.2%). In bivariate analysis, maternal age, parity, gestational age, mode of delivery, and insertion by first‐year residents were associated with device expulsion. In multivariate analysis, maternal age <20 years (aOR 5.03, 95% CI: 1.30–19.42), age 20–34 years (aOR 4.68, 95% CI: 1.40–15.63), and vaginal delivery (aOR 5.78, 95% CI: 2.17–15.42) remained independently associated with IUD expulsion.

**Conclusion:**

At a Brazilian teaching hospital, nearly 15% of the women who had a copper IUD inserted by residents in the immediate postpartum period expelled the device. Maternal age under 35 years and vaginal delivery were significant risk factors for expulsion. These findings highlight the importance of individualized counseling and close follow‐up for higher‐risk groups, as well as continuous training of providers.

## 1 INTRODUCTION

Short interpregnancy intervals and unintended pregnancies remain major global public health challenges associated with increased maternal and perinatal morbidity and mortality.^1^, ^2^ An estimated 60% of unintended pregnancies result in induced abortion, a procedure that remains unsafe in many settings where it is illegal or inaccessible, thereby placing women at risk of complications or death.^3^ In South America alone, approximately four million clandestine abortions are performed annually.^1^

Long‐acting reversible contraceptives (LARCs) play an important role in reducing unintended pregnancies.^4^ Among them, the copper intrauterine device (Cu‐IUD) stands out as a non‐hormonal, safe, low‐cost and highly effective LARC.^5^ Despite its global availability, the use of IUDs in Brazil remains low, with fewer than 2% of women using this method.^6^, ^7^

IUDs can be inserted at various times during a woman's reproductive life, including within the first 10 min after placental delivery—a practice known as postplacental IUD insertion (PP‐IUDI). This timing provides immediate access to a safe and highly effective contraceptive method at a moment when women are often highly motivated to avoid a new pregnancy. PP‐IUDI has several advantages, including confirmed non‐pregnant status, availability of healthcare providers, minimal additional discomfort, and elimination of the need for a return visit for device insertion.^8^ However, these benefits must be weighed against the significantly higher risk of IUD expulsion—estimated to be 7 to 9 times greater than with interval insertion (>4 weeks postpartum).^9^

Reported PP‐IUDI expulsion rates vary widely, ranging from 5% to 50%.^9^, ^10^, ^11^, ^12^, ^13^ This variability may reflect differences in study design, population characteristics, mode of delivery, device type, provider experience, definitions of expulsion, and length of follow‐up.^9^ Brazilian studies reporting Cu‐IUD expulsion after PP‐IUDI describe rates ranging from 8.1% to 36.8%.^12^, ^13^, ^14^, ^15^, ^16^

Since 2015, Cu‐IUDs have been made available free of charge by the Brazilian Ministry of Health to all women who deliver in public maternity hospitals in Brazil. However, there are no national data on expulsion rates following PP‐IUDI in these settings. Hospital‐level estimates of PP‐IUDI expulsion rates and associated risk factors are essential to inform clinical practice, support patient counseling, benchmark institutional performance, and guide quality improvement initiatives. This information is also important to guide evidence‐based strategies to optimize insertion techniques and improve the quality of care provided to women who choose this contraceptive method.

The aim of the present study was to evaluate the incidence of and identify risk factors associated with the expulsion of Cu‐IUDs inserted during the immediate postpartum period in women receiving care at a single public hospital in Brazil. We hypothesized that expulsion rates would be higher in IUDs inserted after vaginal delivery than cesarean sections.

## 2 MATERIALS AND METHODS

### 2.1 Study design, setting, and participants

This retrospective cohort study was carried out at Hospital Municipal Universitário de São Bernardo do Campo (HMUSBC), a public tertiary teaching hospital located in the state of São Paulo, Brazil. Beginning in July 2016, postplacental insertion of T380A‐IUDs was routinely offered, free of charge, to all eligible women admitted for delivery at HMUSBC. Exclusion criteria for PP‐IUDI included current maternal infection, anemia, rupture of membranes for >18 h, or uterine malformation.

We reviewed the medical records of women who delivered at HMUSBC between August 1, 2017, and December 31, 2018 and underwent immediate postpartum insertion of a T380A cooper intrauterine device. Women were included in the analyses if they returned for follow‐up visits up to 12 months after delivery or until documented IUD expulsion. The primary outcomes were the cumulative incidence of IUD expulsion within 12 months of insertion—overall and stratified by mode of delivery—and the identification of risk factors associated with expulsion.

The study was approved by the research ethics committee of São Paulo Federal University (no. 2.905.618) and HMUSBC. Written informed consent was obtained from all participants prior to IUD insertion.

### 2.2 IUD insertion technique

In June 2016, and annually thereafter, all obstetrics and gynecology (OBGYN) residents and on‐duty obstetricians received a brief standardized training on PP‐IUDI techniques after vaginal and cesarean deliveries, led by the principal investigator. For vaginal births, the IUD was inserted through the cervix using a ring forceps up to the highest portion of the uterine cavity while the non‐dominant hand was placed abdominally on the uterine fundus. For cesarean deliveries, the IUD was inserted manually through the hysterotomy incision and guided to the fundus, with the strings directed toward the cervix. In both techniques, the strings were not cut. All insertions were performed by residents under direct supervision of attending obstetricians.

### 2.3 Follow‐up

Before hospital discharge, women received verbal instructions from nursing staff regarding IUD expulsion signs and potential complications (e.g., infection, pain, bleeding) and were advised to seek immediate medical attention at the hospital's emergency department if any concerns arose. Before leaving the hospital, all women were scheduled for a routine transvaginal ultrasonography (TVUSG) approximately 40 days later. If the sonogram confirmed that the IUD was located inside the uterine cavity, above the internal cervical orifice, the woman was scheduled for two additional follow‐up visits at 6 and 12 months after delivery. At these visits, an OBGYN resident documented gynecologic symptoms, breastfeeding status, and conducted speculum and pelvic examinations. If IUD strings were not visible, a new TVUSG was performed to confirm device location.

IUD expulsion was diagnosed based on the following criteria: (i) self‐reported expulsion at home, (ii) IUD not visualized on TVUS, (iii) IUD seen in the vagina on speculum examination, (iv) IUD located in the cervical canal on TVUSG or seen protruding through the external cervical orifice on speculum examination, (v) distal end of the IUD shaft below the internal cervical orifice on TVUSG. Expulsions were classified as complete (situations i, ii, and iii) or partial (situations iv and v). Women with partial expulsions had the device removed.

### 2.4 Data extraction and analysis

Demographic, obstetric, and clinical data were extracted from medical records using a structured data collection form and entered into an electronic database for analysis. We calculated the cumulative incidence of IUD expulsions and corresponding 95% confidence interval (CI) for the entire cohort and by mode of delivery. We conducted univariate and multivariate analyses to examine the association between maternal, pregnancy and delivery characteristics (independent variables) and device expulsion (dependent variable). Eight variables were included in the univariate analysis, based on the literature and clinical experience: maternal age, self‐reported skin color, parity, gestational age at delivery, labor induction, mode of delivery, type of healthcare provider performing insertion, and breastfeeding status (at 12 months or at the time of expulsion). Variables with a *P* value less than 0.21 in univariate analysis (Chi‐square or Fisher exact tests) were entered in the multivariate logistic regression analysis (Wald method) to identify independent risk factors for expulsion. A two‐tailed *P* value less than 0.05 was considered statistically significant. Crude and adjusted odds ratio (OR) with 95% CI are reported for each variable. All statistical analyses were performed using STATA17 (StataCorp LP, College Station, Texas, USA).

## 3 RESULTS

During the study period, 8043 women delivered at HMUSBC and 1718 (21.4%) underwent PP‐IUDI of a Cu‐IUD. Of these, 543 women (31.6%) returned for follow‐up visits up to 12‐months postpartum or until documented IUD expulsion, and were included in the final analyses.

Participant ages ranged from 14 to 46 years. Most lived with a partner (71.1%), self‐identified as non‐white (57.8%), had ≥12 years of education (73.7%), were multiparous (70.9%), and delivered vaginally (54.1%) (Table 1). A total of 80 women expulsed the IUD within the first 12 months post‐insertion, yielding an overall cumulative expulsion rate of 14.7% (95% CI: 12.0%–18.0%). The incidence of expulsion was significantly higher among women who delivered vaginally than by cesarean section: 22.8% (95% CI: 17.7%–28.9%) versus 5.2%, (95% CI: 2.8%–8.9%), respectively. Most of the cases (62.5%, *n* = 50) were classified as complete expulsions. A total of 50% (*n* = 40) of the expulsions occurred within the first 6 weeks postpartum, 42.5% (*n* = 34) occurred between 7 weeks and 6 months, and 7.5% (*n* = 6) occurred after 6 months.

**TABLE 1:** Main characteristics of 543 women with post‐placental copper IUD insertion at one public hospital in Sao Paulo, Brazil.

Characteristic	
Age, years	
Mean (SD)	26.3 (6.7)
<20	91 (16.7)
20–34	374 (68.9)
35–40	65 (12.0)
>40	13 (2.4)
Marital status	
Married	151 (27.8)
Common‐law marriage	235 (43.3)
Single	157 (28.9)
Self‐reported skin color (*n* = 540)	
Mixed	295 (54.6)
White	228 (42.2)
Black	14 (2.6)
Yellow	3 (0.6)
Education, years	
<12	143 (26.3)
12	359 (66.1)
>12	41 (7.6)
Parity	
Primipara^a^	158 (29.1)
Multipara	385 (70.9)
Model of delivery	
Vaginal	294 (54.1)
Cesarean	249 (45.9)

Abbreviations: IUD, intrauterine device; SD, standard deviation.

^a^ This was the woman's first delivery.

In the univariate analysis, younger maternal age, vaginal delivery and insertion by first‐year residents were identified as significant risk factors for IUD expulsion (Table 2). These variables, along with parity and gestational age at delivery (*P* < 0.21) were included in the multivariate analysis. In the final multivariate model, only maternal age and mode of delivery remained independently associated with IUD expulsion. Compared to women aged ≥35 years, those aged <20 years had a five‐fold increased risk of IUD expulsion (aOR 5.03, 95% CI: 1.30–19.42, *P* = 0.019), while women aged 20–34 years had a 4.68‐fold higher risk (aOR 4.68, 95% CI: 1.40–15.63, *P* = 0.012). Additionally, women who delivered vaginally were nearly six times more likely to expel the IUD than those who had a cesarean delivery (aOR 5.78, 95% CI: 2.17–15.42, *P* < 0.001). Gestational age at delivery <37 weeks showed a trend toward significance (aOR 2.25; 95% CI: 0.95–5.37; *P* = 0.067). Primiparity and IUD insertion by first‐year residents were not significantly associated with device expulsion in the multivariate analysis. (Tables 2 and 3).

**TABLE 2:** Risk factors for IUD expulsion among 543 women with immediate postpartum device insertion.

Variable	Total *N*	Expulsion	No expulsion	cOR	95% CI	*P* value	aOR	95% CI	*P* value
Maternal age, years						0.009^a^			0.041
<20	91	18 (19.8)	73 (80.2)	6.16	(1.74; 21.82)		5.03	(1.30; 19.42)	
20–34	374	59 (15.8)	315 (84.2)	4.68	(1.43;15.35)		4.68	(1.40; 15.63)	
≥35 (ref)	78	3 (3.8)	75 (96.2)	1.00	Reference		1.00	Reference	
Skin color (*n* = 540)						0.874			
Non‐White	312	45 (14.4)	267 (85.6)	0.96	(0.59; 1.56)				
White (ref)	228	34 (14.9)	194 (85.1)	1.00	Reference				
Parity						0.208^a^			0.889
Primipara	158	28 (17.7)	130 (82.3)	1.38	(0.82; 2.28)		1.05	(0.56; 1.95)	
Multipara (ref)	385	52 (13.5)	333 (86.5)	1.00	Reference		1.00	Reference	
Gestational age at delivery						0.175^a^			0.067
<37 weeks	41	9 (22.0)	32 (78.0)	1.71	(0.78; 3.73)		2.25	(0.95; 5.37)	
≥37 weeks (ref)	502	71 (14.1)	431 (85.9)	1.00	Reference		1.00	Reference	
Mode of delivery						<0.001^a^			<0.001
Vaginal	294	67 (22.8)	227 (77.2)	5.36	(2.88; 9.97)		5.78	(2.17; 15.42)	
Cesarean (ref)	249	13 (5.2)	236 (94.8)	1.00	Reference		1.00	Reference	
Provider inserting IUD						0.002^a^			0.623
First year resident	422	73 (17.3)	349 (82.7)	3.41	(1.52; 7.61)		1.35	(0.41; 4.51)	
Second/third year resident (ref)	121	7 (5.8)	114 (94.2)	1.00	Reference		1.00	Reference	
Induced labor						0.462			
Yes	126	16 (12.7)	110 (87.3)	0.80	(0.45; 1.44)				
No (ref)	417	64 (15.3)	353 (84.7)	1.00	Reference				
Breastfeeding status						0.954			
Yes	212	31 (14.6)	181 (85.4)	0.99	(0.61; 1.60)				
No (ref)	331	49 (14.8)	282 (85.2)	1.00	Reference				

*Note*: All values represent numbers (%) unless otherwise stated.

Abbreviations: aOR, adjusted odds ratio; CI, confidence interval; cOR, crude odds ratio; IUD, intrauterine device.

^a^ Variables included in multivariate logistic regression.

**TABLE 3:** Multivariate logistic regression analysis of IUD expulsion risk factors.

	Regression coefficient (β)	SE	Wald *X* ^2^	*P* value	aOR	95% CI
Age category, years			6.409	0.041		
<20	1.615	0.690	5.482	0.019	5.03	(1.30; 19.42)
20–34	1.543	0.616	6.277	0.012	4.68	(1.40; 15.63)
Primipara	0.045	0.318	0.020	0.889	1.05	(0.56; 1.95)
Gestational age < 37 weeks	0.813	0.443	3.358	0.067	2.25	(0.95; 5.37)
Vaginal delivery	1.755	0.501	12.289	<0.001	5.78	(2.17; 15.42)
Insertion by first year resident	0.302	0.615	0.242	0.623	1.35	(0.41; 4.51)
Constant	−4.478	0.764	34.379	<0.001	0.011	—

*Note*: Reference categories: ≥ 35 years, multiparity, gestational ≥37 weeks, cesarean delivery, second or third year resident.

Abbreviations: aOR, adjusted odds ratio; CI, confidence interval; IUD, intrauterine device; OR, odds ratio; SE, standard error.

## 4 DISCUSSION

In the present study we found an overall PP‐IUDI expulsion rate of 14.7% within the first year of follow‐up, with significantly higher rates for IUDs inserted after vaginal deliveries (22.8%) compared to cesarean deliveries (5.2%). In the multivariate analysis, maternal age under 35 years and insertion after vaginal delivery were identified as independent risk factors for IUD expulsion.

Our findings are consistent with the existing literature on overall immediate PP‐IUDI expulsion rates, and higher rates for insertions after vaginal deliveries.^14^, ^17^ According to the most recent systematic review on this topic, the average rate of complete expulsions for IUDs (any model) inserted in the immediate postpartum period was 9.1% (95% CI: 0%–25.4%), based on 13 studies (*n* = 2213 women) that had follow‐up periods >6 months.^9^ For Cu‐IUDs, the average complete expulsion rate for IUDs inserted after vaginal delivery was 13.0% (95% CI: 4.8%–23.5%, 4 studies, 1026 women) and for insertions after cesareans, the expulsion rate was 4.0% (95% CI: 2.0%–15.0%, 5 studies, 817 women).^9^ Although our expulsion rates were somewhat higher for vaginal and cesarean deliveries (22.8% and 5.2%, respectively), they are within the reported CIs of that systematic review. Possible explanations for our higher expulsion rates include our longer follow‐up period, our inclusion of partial expulsions, and the fact that all IUDs were inserted by residents, which may reflect lower levels of procedural experience. Previous studies have shown that provider training and experience significantly influence postplacental IUD expulsion rates.^18^, ^19^ The association between younger maternal age and higher expulsion risk has been reported by other researchers and may be related to smaller uterine dimensions and increased uterine tone in younger women.^11^, ^16^, ^20^ However, contrary to the findings of a large cohort study that analyzed risk factors for PP‐IUD expulsions,^10^ we found no significant association between maternal ethnicity or breastfeeding status and the risk of IUD expulsion.

The strengths of this study include its large sample size, the analysis of several potential risk factors for IUD expulsion, and its length of follow‐up, making it the largest cohort from Brazil examining PP‐IUD expulsion up to the 12th month after delivery.^12^, ^13^, ^14^, ^15^, ^16^, ^21^ However, we acknowledge several limitations, such as the retrospective study design, reliance on medical records for data collection, and the high attrition rate in follow‐up visits after IUD insertion. Non‐adherence to postpartum visits is common in Brazilian women in general.^22^, ^23^ Moreover, several international studies report that less than 50% of women, especially from low‐ and middle‐income countries, return for follow‐up visits after PP‐IUDI.^17^, ^24^, ^25^ Potential reasons for non‐adherence to follow‐up visits in our study include lack of understanding of discharge instructions, socioeconomic and financial constraints, difficulties in rescheduling missed appointments, and women's prioritization of newborn care over their own healthcare in the first year after delivery. Finally, the single center setting and exclusive involvement of resident physicians may limit the generalizability of our findings to other institutions or healthcare settings.

Based on the results of this study, we reinforced the training for OBGYN residents especially for PP‐IUDI after vaginal delivery. Now, the training consists of a simulation‐based session followed by a supervised clinical demonstration. Additionally, discharge counseling was strengthened to emphasize the importance of follow‐up visits after PP‐IUDI, especially for younger women and those who had a vaginal delivery. The effects of these interventions will be described in an upcoming publication. Plans are underway to conduct telephone interviews with the women who miss scheduled follow‐up appointments to understand what are the alleged reasons, and to design strategies to improve PP‐IUDI surveillance. Further research is needed to identify additional risk factors for PP‐IUDI expulsion, and strategies to improve follow‐up adherence, especially for high‐risk women in settings with limited healthcare resources.

In conclusion, the cumulative 12‐month expulsion rate of Cu‐IUDs inserted by OBGYN residents in the immediate postpartum period was approximately 15%, with significantly higher expulsion rates for devices inserted after vaginal than after cesarean delivery. Maternal age under 35 years and insertion after vaginal delivery were independently associated with increased risk of IUD expulsion. These findings highlight the importance of individualized counseling and close follow‐up of women at higher risk of IUD expulsion, as well as continuous training of providers.

## AUTHOR CONTRIBUTIONS

**Silvana A. Giovanelli:** Conceptualization, data curation, investigation, writing – original draft, review and editing. **Maria Regina Torloni:** Data curation, methodology, formal analysis, investigation, writing – original draft, review and editing. **Cristina A. F. Guazzelli:** Conceptualization, data curation, methodology, supervision, writing – review and editing. **Silvana A. Giovanelli, Maria Regina Torloni and Cristina A. F. Guazzelli:** Approved the final version to be published and agree to be accountable for all aspects of the work in ensuring that questions related to the accuracy or integrity of any part of the work are appropriately investigated and resolved.

## CONFLICT OF INTEREST STATEMENT

The authors have no conflicts of interest.

## Data Availability

Research data are not shared.

## References

[1] Langer A . Unwanted pregnancy: impact on health and society in Latin America and the Caribbean. Revista Panamericana de Salud Publica = Pan Am J Public Health. 2002;11(3):192‐204. 10.1590/s1020-49892002000300013 11998185

[2] Ahrens KA , Nelson H , Stidd RL , Moskosky S , Hutcheon JA . Short interpregnancy intervals and adverse perinatal outcomes in high‐resource settings: an updated systematic review. Paediatr Perinat Epidemiol. 2019;33(1):O25‐O47. 30353935 10.1111/ppe.12503 PMC7379643

[3] Bearak J , Popinchalk A , Ganatra B , et al. Unintended pregnancy and abortion by income, region, and the legal status of abortion: estimates from a comprehensive model for 1990‐2019. Lancet Glob Health. 2020;8(9):e1152‐e1161. 32710833 10.1016/S2214-109X(20)30315-6

[4] Wu JP , Moniz MH , Ursu AN . Long‐acting reversible contraception‐highly efficacious, safe, and underutilized. JAMA. 2018;320(4):397‐398. 29984374 10.1001/jama.2018.8877 PMC13255570

[5] World Health Organisation . Medical Eligibility Criteria for Contraceptive Uses, Fifth Edition [WHO website]. 2015 Accessed May 12, 2025. https://www.who.int/publications/i/item/9789241549158

[6] de Ponce Leon RG , Ewerling F , Serruya SJ , et al. Contraceptive use in Latin America and the Caribbean with a focus on long‐acting reversible contraceptives: prevalence and inequalities in 23 countries. Lancet Glob Health. 2019;7(2):e227‐e235. 30683240 10.1016/S2214-109X(18)30481-9 PMC6367565

[7] Buhling KJ , Zite NB , Lotke P , Black K , INTRA Writing Group . Worldwide use of intrauterine contraception: a review. Contraception. 2014;89(3):162‐173. 24369300 10.1016/j.contraception.2013.11.011

[8] Makins A , Ahsan A , Waqas M , et al. FIGO position statement on postpartum intrauterine devices (PPIUD). Int J Gynaecol Obstet. 2025;169(3):1127‐1132. 40285713 10.1002/ijgo.70146 PMC12093926

[9] Averbach SH , Ermias Y , Jeng G , et al. Expulsion of intrauterine devices after postpartum placement by timing of placement, delivery type, and intrauterine device type: a systematic review and meta‐analysis. Am J Obstet Gynecol. 2020;223(2):177‐188. 32142826 10.1016/j.ajog.2020.02.045 PMC7395881

[10] Armstrong MA , Raine‐Bennett T , Reed SD , et al. Association of the Timing of postpartum intrauterine device insertion and breastfeeding with risks of intrauterine device expulsion. JAMA Netw Open. 2022;5(2):e2148474. 35226086 10.1001/jamanetworkopen.2021.48474 PMC8886522

[11] Keenahan L , Bercaw‐Pratt JL , Adeyemi O , Hakim J , Sangi‐Haghpeykar H , Dietrich JE . Rates of intrauterine device expulsion among adolescents and young women. J Pediatr Adolesc Gynecol. 2021;34(3):362‐365. 33189897 10.1016/j.jpag.2020.11.003

[12] Marangoni M Jr , Laporte M , Surita F , Kraft MB , Bahamondes L , Juliato CRT . One‐year follow up on post‐placental IUD insertion: a randomized clinical trial. Acta Obstet Gynecol Scand. 2021;100(4):596‐603. 33421091 10.1111/aogs.14081

[13] Nahas G , Magalhaes C , Bueloni‐Dias F , Nahas E , Borges V . Immediate postpartum insertion of copper intrauterine device in a Brazilian university hospital: expulsion and continuation rates. Rev Bras Ginecol Obstet. 2023;45(1):31‐37. 36878250 10.1055/s-0042-1759628 PMC10021007

[14] Hochmuller JT , Lopes KS , Guazzelli CAF , Gomes MKO , Araujo JE , Peixoto AB . Expulsion rate of intrauterine device: mediate vs. immediate puerperium period. J Turk Ger Gynecol Assoc. 2020;21(3):143‐149. 32517434 10.4274/jtgga.galenos.2020.2020.0037 PMC7495121

[15] da Silva Nobrega AB , Pitangui ACR , Vieira CS . Factors associated with missing strings and expulsion after postplacental insertion of copper T380A intrauterine devices. Int J Gynaecol Obstet. 2022;157(1):67‐75. 34197636 10.1002/ijgo.13806

[16] Giovanelli SA , Torloni MR , Guazzelli CAF . Post‐placental intrauterine device insertion in Brazilian adolescents: clinical outcomes at 12 months. J Pediatr Adolesc Gynecol. 2022;35(3):336‐340. 34737030 10.1016/j.jpag.2021.10.009

[17] Hooda R , Mann S , Nanda S , Gupta A , More H , Bhutani J . Immediate postpartum intrauterine contraceptive device insertions in caesarean and vaginal deliveries: a comparative study of follow‐up outcomes. Int J Reprod Med. 2016;2016:7695847. 27631023 10.1155/2016/7695847 PMC5005603

[18] Cole M , Thomas S , Mercer BM , Arora KS . Impact of training level on postplacental levonorgestrel 52 mg intrauterine device expulsion. Contraception. 2019;99(2):94‐97. 30452904 10.1016/j.contraception.2018.11.003 PMC6351181

[19] Cooper M , McGeechan K , Glasier A , et al. Provision of immediate postpartum intrauterine contraception after vaginal birth within a public maternity setting: health services research evaluation. Acta Obstet Gynecol Scand. 2020;99(5):598‐607. 31837002 10.1111/aogs.13787 PMC7217220

[20] Anthony MS , Zhou X , Schoendorf J , et al. Demographic, reproductive, and medical risk factors for intrauterine device expulsion. Obstet Gynecol. 2022;140(6):1017‐1030. 36357958 10.1097/AOG.0000000000005000 PMC9665953

[21] Zaconeta AM , Oliveira AC , Estrela FS , et al. Intrauterine device insertion during cesarean section in women without prenatal contraception counseling: lessons from a country with high cesarean rates. Rev Bras Ginecol Obstet. 2019;41(8):485‐492. 31450255 10.1055/s-0039-1693677 PMC10316793

[22] Silva CS , Schuster RV , Dornfeld D , Patuzzi GC , Szczepanik JC , Neutzling AL . Follow‐up visit after post‐placental copper intrauterine device insertion (TCu 380A). Cienc Cuid Saude. 2023;22:e64631.

[23] Baratieri T , Lentsck MH , Falavina LP , Soares LG , Prezotto KH , Pitilin EB . Longitudinal care: factors associated with adherence to postpartum follow‐up according to data from PMAQ‐AB. Cad Saude Publica. 2022;38(3):e00103221. 35293537 10.1590/0102-311X00103221

[24] Makins A , Taghinejadi N , Sethi M , et al. FIGO postpartum intrauterine device initiative: complication rates across six countries. Int J Gynaecol Obstet. 2018;143(Suppl 1):20‐27. 30225873 10.1002/ijgo.12600

[25] Levi E , Cantillo E , Ades V , Banks E , Murthy A . Immediate postplacental IUD insertion at cesarean delivery: a prospective cohort study. Contraception. 2012;86(2):102‐105. 22264666 10.1016/j.contraception.2011.11.019

